# Editorial: Rising stars in ethnopharmacology: 2021

**DOI:** 10.3389/fphar.2023.1224820

**Published:** 2023-06-07

**Authors:** Ulrike Grienke, Francesca Scotti, Judith M. Rollinger

**Affiliations:** ^1^ Division of Pharmacognosy, Department of Pharmaceutical Sciences, Faculty of Life Sciences, University of Vienna, Vienna, Austria; ^2^ School of Pharmacy, Faculty of Life Sciences, University College London, London, United Kingdom

**Keywords:** ethnopharmacology, *Momordica charantia*, *Entada africana*, alkannin, palmatine, skin disorders, diterpene resin acids, bilberries

This Research Topic wishes to promote the high quality and diverse work of internationally recognized and up-and-coming researchers in the earlier stages of their career in the field of ethnopharmacology. The Research Topic provides a dedicated platform for these researchers to present and discuss their findings, with the purpose of highlighting novel outcomes and opening up routes for future directions in the broad field of ethnopharmacology.

The four original articles and the three reviews collected offer a potpourri of highlights on the stunning diversity of topics within ethnopharmacology. The original articles present pharmacological results (*in vitro* and *in vivo*), as well as analytical studies (for quality control) investigating extracts and thereof isolated compounds for the rationalization of the traditional use of herbal drugs. The rising stars in ethnopharmacology include authors from different countries/continents including Greece, Austria, Germany, Ghana, and Benin, thereby reflecting the longstanding popularity and tradition of the ethnopharmacological use of herbal drugs in the respective countries.

The ethnopharmacological use of *Momordica charantia* L. (Cucurbitaceae), the bitter gourd or bitter melon, its constituents (in particular cucurbitane-type triterpenoids) and their pharmacological effects against diabetes-related conditions, as well as potential adverse effects, are the topic of the mini review by Çiçek. The author highlighted the need to make a distinction between different diabetes-related activities, including unspecified hypoglycemic effects, compounds enhancing glucose uptake, targeting glucose absorption or production, stimulating insulin secretion, and compounds targeting insulin resistance. In addition, aspects of quality control and issues of standardization of commercial products are discussed.

Tsioutsiou et al. reviewed the ethnopharmacological application of medicinal plants against skin diseases in the South Balkan and East Mediterranean region. In their work, the authors extracted relevant information out of more than 120 ethnobotanical studies, including a detailed statistical analysis regarding the taxonomic distribution of used plant species over more than 100 families. Concerning the application of the plant materials against skin disorders, the authors distinguished between internal and external use and also listed specific indications reported in the investigated studies, such as burns, hemorrhoids, or furuncles. The work concludes that in comparing the obtained results with Dioscorides’ “*De Materia Medica*,” a close connection and persistence in the use of plants against skin disorders can be found in the whole geographic area.

Bilberries, the fruits of *Vaccinium myrtillus*, ingredients for local foods and medicine, have long been associated with supporting vision and with claims of helping in the management of sugar levels and cardiovascular disorders. In their paper, Vaneková et al. review in detail the species’ known rich chemistry and the scientifically investigated uses, offering a short overview that helps debunking its unsupported claims while bringing to light its updated medicinal applicability. While there are insufficient studies for the application of bilberry fruits for patients suffering from diabetes and impaired night vision, there is sufficient evidence to prove the bilberries’ anti-inflammatory activity, backed up by clinical trials, at least for the application in cases of gingivitis and bowel inflammation. Interestingly, the use for circulatory disorders (approved by EMA) is supported, but the studies are outdated and not up to current clinical trial standards.

The original article by Codo Toafode et al. highlights the *in vitro* anti-inflammatory activity of the West African plant *Entada africana* Guill. & Perr. (Fabaceae). Using a bioactivity-guided fractionation approach, eleven phenolic constituents were isolated from three fractions of the hydroalcoholic leaf extract. To rationalize the traditional use of the species in the Republic of Benin, an *in vitro* model for skin inflammation using TNF-α stimulated human keratinocytes (HaCaT) was applied. The results show the release of proinflammatory cytokines IL-8 and IL-6, revealing strong to medium activities for some of the isolated constituents.

In another work, the anti-cancer properties of the protoberberine alkaloid palmatine were investigated by Ativui et al. Interestingly, the compound is an active component in different traditional African herbal preparations. Beside its antimetastatic and antiproliferative effects, palmatine was found to inhibit metastatic colonization of triple negative breast cancer cells in the lungs by simultaneously preserving lung morphology.

Arampatzis et al. investigated a set of hydroxynaphthoquinone compounds including alkannin, shikonin and their derivatives known as constituents of Boraginaceae plants for anti-obesity/insulin-mimetic properties as well as for anthelmintic effects. Two model systems were used for testing, i.e., 3T3-L1 pre-adipocytes and the nematode *Caenorhabditis elegans*. By analyzing the obtained data, valuable structure-activity-relationships could be determined showing that structural variations significantly impact biological activities in both model systems investigated in this study.

The work of Goels et al. tackles the analytical challenges of Norway spruce balm. The exudate, produced by *Picea abies*, is mainly composed of diterpene resin acids (DRAs) which are knowingly tricky to separate analytically. Norway spruce balm is referenced in the Austrian Pharmacopoeia (2021 ed.) but the monograph currently lacks both rigorous quality control methods for marker compounds and the minimum content for any compound. Considering DRAs constitute the main active component of the balm, their content determination would be pivotal for identifying the quality of any Norway spruce balm product. This is why the authors developed an Ultra-High-Performance Supercritical Fluid Chromatography method that optimizes the analyses of these compounds, overcoming the inefficiency, unsustainability and lengthy times of conventional liquid and gas chromatography protocols. The analysis of twenty-two Norway spruce balm samples and products showed the efficiency and selectivity of the developed protocol. Additionally, the method is more ecological, consuming significantly less solvents than other chromatographic methodologies.

In conclusion, the Research Topic contains a diverse selection of studies highlighting the relevance and applicability of ethnopharmacology not only for science but also for society.

